# Intelligent Informatics in Translational Medicine 2016

**DOI:** 10.1155/2017/1572730

**Published:** 2017-02-28

**Authors:** Hao-Teng Chang, Tatsuya Akutsu, Oliver Ray, Sorin Draghici, Tun-Wen Pai

**Affiliations:** ^1^Graduate Institute of Basic Medical Science, College of Medicine, China Medical University, Taichung, Taiwan; ^2^Department of Computer Science and Information Engineering, Asia University, Taichung, Taiwan; ^3^Department of Science Education, Affiliated Dongyang Hospital of Wenzhou Medical University, Dongyang, Zhejiang, China; ^4^Bioinformatics Center, Institute for Chemical Research, Kyoto University, Kyoto, Japan; ^5^Department of Computer Science, University of Bristol, Bristol BS8 1UB, UK; ^6^Department of Computer Science, Wayne State University, Detroit, MI 48202, USA; ^7^Department of Computer Science and Engineering, National Taiwan Ocean University, Keelung, Taiwan; ^8^Center of Excellence for the Oceans, National Taiwan Ocean University, Keelung, Taiwan

This special issue is the second annual special issue of Intelligent Informatics in Translational Medicine (IITM). Only seven papers were published in this special issue after rigorous reviewing processes, of which four papers were selected from the 10th International Conference on Systems Biology (ISB 2016) organized by Computational Systems Biology Society of ORSC (China) in August 2016.

In 2013, we have launched the first special issue with high-quality original research articles, and the title of the special issue was named as Intelligent Informatics in Biomedicine. Most of the published papers were recommended by the International Workshop on Intelligent Informatics in Biology and Medicine (IIBM). In order to extend the scope of research areas and continue this fruitful discussion, we renamed the special issue as the new title of Intelligent Informatics in Translational Medicine (IITM) in 2014 and organized this special issue as a series edition for discovering more biological insights from genomics data or any medical big data. This special issue focuses on the challenges and solutions for information processes with an emphasis on forthcoming high throughput technology and systems biomedicine.

Advances in information technologies are facilitating and accelerating research on molecular biology and medicine. Especially in the precision-medicine era, the increasing complexity and volume of biological data mean that more sophisticated computational techniques are urgently required. Such methods must be able to support new sensing techniques that are being developed to improve the quality of healthcare and medicine. The use of artificial intelligence, machine learning, and data mining can potentially play a significant role in addressing these important challenges. We hope the published series special issue will provide an opportunity for academic and industry professionals to discuss the latest issues and progress in the area of biomedicine.

The current special issue aims to combine intelligent informatics and bioinformatics on diseases and translation medicine, and these collected papers address the data-analytical method design, algorithm development, mathematical modeling, and computational simulation techniques to the translational medical applications.

X. Jiang et al. in their article entitled “Differentially Coexpressed Disease Gene Identification Based on Gene Coexpression Network” designed a novel framework to identify disease-related genes and developed a differentially coexpressed disease-related gene identification method based on gene coexpression network to screen differentially coexpressed genes. They constructed phase-specific gene coexpression network using time-series gene expression data and defined the conception of differential coexpression of genes in coexpression network. They also designed two metrics to measure the value of gene differential coexpression according to the change of local topological structures between different phase-specific networks. At last, they performed meta-analysis of gene differential coexpression based on a rank-product approach. Their experimental results have shown the feasibility and effectiveness of such a gene coexpression network and the superior performance over other popular disease-related gene selection approaches.

Y. Su et al. described that oncolytic virus is a kind of tumor killer virus which can infect and lyse cancer cells and spread through the tumor, while leaving normal cells largely unharmed. We know that appropriate mathematical models could help biologists to understand the tumor-virus dynamics and find better treatment strategies. The authors proposed a new mathematical model of tumor therapy with oncolytic virus and MEK inhibitor. Due to mitogen-activated protein kinase providing greater oncolytic virus infection into cancer cells and limiting the replication of the virus, in order to provide the best dosage of MEK inhibitors and balance the positive and negative effects of the inhibitors, authors proposed an optimal control strategy regarding the inhibitor. Simulations have shown that the optimal control indeed possessed better control effects than constant control under the same initial conditions.

C. Quan et al. proposed a multichannel convolutional neural network for automated biomedical relation extraction. The proposed model provides two contributions: it enables the fusion of multiple versions in word embedding and the need for manual feature engineering can be obviated by automated feature learning with convolutional neural network. The authors have evaluated the proposed model on two biomedical relation extraction tasks including drug-drug interaction extraction and protein-protein interaction extraction. For conducting several experimental trials on benchmark testing datasets, the proposed system outperformed the standard SVM based systems within a range from 2.7% to 5.6% on *F*-score measurement.

Y. X. Goay et al. in their article entitled “Identification of Five Novel* Salmonella* Typhi-Specific Genes as Markers for Diagnosis of Typhoid Fever Using Single-Gene Target PCR Assays” identified new markers to detect the* Salmonella* Typhi pathogen and developed sensitive and specific diagnostic tests in this study. Based on genomic comparison of* Salmonella* Typhi with other enteric pathogens, there are six* Salmonella* Typhi genes found to be specific, and corresponding PCR assays for each target gene were developed to verify their specificity and sensitivity in vitro. The experimental results showed that 5 genes selected from the 6 candidate genes could be demonstrated with perfect verification results (100% sensitivity and 100% specificity), and these genes could be applied as important biomarkers for diagnosis of typhoid fever through single-gene target PCR-assays.

N. Yu et al. proposed a single-trial sparse representation-based approach for visual evoked potential (VEP) extraction. Sparse representation is a powerful tool in signal denoising, and visual evoked potentials have been proven to have strong sparsity over an appropriate dictionary. The authors presented such a novel sparse representation-based approach to solving the extraction problem. Three stages were proposed in their article: utilizing the previous electroencephalogram (EEG) data to identify the parameters of the EEG autoregressive (AR) model; applying sparse representation to model the VEPs in the autoregressive-moving average model; calculating the sparse coefficients and deriving VEPs by using the AR model. The proposed method was verified and compared by employing synthetic and real data against different existing methods, and the evaluation demonstrates that their proposed method can well preserve the details of the VEPs for latency estimation, even in low SNR environments.

B. Tang developed a toolkit META2 for DNA methylation annotation and analysis. The tool performs integrative analysis on differentially methylated loci and regions through deep mining and statistical comparison methods. The author examined the association within differentially methylated CpG and differentially methylated region candidates regarding counts and region lengths and identified major transition zones as clues for inferring statistically significant regions. The developed tool can provide a comprehensive analysis approach for epigenetic research and clinical study.

Y. Li et al. constructed a multilevel evolutionary structure for avian influenza virus system based on considering both hemagglutinin and neuraminidase protein fragments. An optimization model was established to determine the rational granularity of the virus system for exploring the intrinsic relationship among the subtypes based on the fuzzy hierarchical evaluation index. To reduce the systematic and computational complexity of the proposed algorithm, the granular signatures of virus system were identified based on the coarse-grained idea. The proposed system can effectively identify the virus signatures and reflect the whole avian influenza virus system, which indicates that the proposed method provides an alternative mechanism to perform new virus subtyping comparison and functional prediction.

